# The use of stretching devices for treatment of trismus in head and neck cancer patients: a randomized controlled trial

**DOI:** 10.1007/s00520-019-05075-7

**Published:** 2019-11-07

**Authors:** Sarah J. van der Geer, Harry Reintsema, Jolanda I. Kamstra, Jan L. N. Roodenburg, Pieter U. Dijkstra

**Affiliations:** 1 Department of Oral and Maxillofacial Surgery, University of Groningen, University Medical Center Groningen, Hanzeplein 1, 9713 GZ Groningen, the Netherlands; 2 Department of Rehabilitation, University of Groningen, University Medical Center Groningen, Hanzeplein 1, 9713 GZ Groningen, the Netherlands

**Keywords:** Head and neck neoplasms, Mouth neoplasms, Trismus, Quality of life, Rehabilitation, Exercise therapy

## Abstract

**Purpose:**

To compare the effects of two stretching devices, the TheraBite® Jaw Motion Rehabilitation System™ and the Dynasplint Trismus System®, on maximal mouth opening in head and neck cancer patients.

**Methods:**

Patients were randomly assigned to one of two exercise groups: the TheraBite® Jaw Motion Rehabilitation System™ group or the Dynasplint Trismus System® group. Patients performed stretching exercises for 3 months. During the three study visits, maximal mouth opening was measured and the patients completed questionnaires on mandibular function and quality of life.

**Results:**

In our study population (*n* = 27), five patients did not start the exercise protocol, eight patients discontinued exercises, and two patients were lost to follow-up. No significant differences regarding the change in mouth opening between the two devices were found. Patients had an increase in MMO of 3.0 mm (IQR − 2.0; 4.0) using the TheraBite® Jaw Motion Rehabilitation System™ and 1.5 mm (IQR 1.0; 3.0) using the Dynasplint Trismus System®. Exercising with either stretching device was challenging for the patients due to the intensive exercise protocol, pain during the exercises, fitting problems with the stretching device, and overall deterioration of their medical condition.

**Conclusions:**

The effects of the two stretching devices did not differ significantly in our study population. The factors described, influencing the progression of stretching exercises, need to be taken into account when prescribing a similar stretching regimen for trismus in head and neck cancer patients.

**Trial registration:**

NTR - Dutch Trial Register number: 5589

**Electronic supplementary material:**

The online version of this article (10.1007/s00520-019-05075-7) contains supplementary material, which is available to authorized users.

## Introduction

To treat trismus in head and neck cancer patients, a variety of exercise therapies are prescribed [1]. Exercise therapy with stretching devices such as the TheraBite® Jaw Motion Rehabilitation System™ (TheraBite) and the Dynasplint Trismus System® (DTS) have been reported to increase mouth opening up to 14 mm [2–4]. However, the effects of these two devices have not been compared side-by-side. In a randomized controlled trial, we compared the effects of the TheraBite and the DTS on mouth opening in head and neck cancer patients with trismus.

## Materials and methods

Patients were eligible if they were at least 18 years old and if they were treated for head and neck cancer and had a maximal mouth opening (MMO) of 35 mm or less. Based on a sample size calculation, 24 patients per group were needed. To compensate for drop-outs, we aimed to include 30 patients per group. The patients were randomized on the basis of two strata, with blocks of four with an allocation ratio of one. One stratum included patients who received cancer treatment less than 36 months ago, and the other included patients who received cancer treatment 36 months ago or longer.

During the first visit (T1), the stretching device and a diary were provided. During all three study visits, patients filled in questionnaires regarding mandibular function and quality of life and their MMO was measured. An interview was held about patients’ experiences with the stretching devices and the exercise protocol, during the last two visits (T2, T3). More details can be found in Supplementary Material Text 1.

## Results

In total, 166 patients were assessed for eligibility, of which 86 did not meet the inclusion criteria, 35 declined to participate, and 18 did not participate due to other reasons. Patients declined to participate because they thought the protocol was too intensive or because they had no treatment need. Other reasons for non-participation were overall poor health, old age (≥ 80 years), or because using the stretching device was not possible due to previous extensive oncological treatment (such as hemi-mandibulectomy or large cheek resections). Of the 27 patients who were eventually enrolled in the study, five patients did not start the exercise program due to reconsideration of the exercise program (*n* = 2; too intensive, too much comorbidity), occurrence or suspicion of new tumor (*n* = 2), and because the use of a stretching device was not possible (*n* = 1; high sensitivity of the lower jaw). Of the 22 patients who started the protocol, eight discontinued exercises due to depression (*n* = 1), painful mouth/neck (*n* = 3), recurrent tumor (*n* = 1), overly intensive protocol (*n* = 2), and the use of stretching device was not possible (*n* = 1) (MMO of 7 mm). Two patients were lost to follow-up.

Eleven adverse events occurred (Supplementary Material Text 2). Four serious adverse events were reported, but were unrelated to the stretching device (metastasis (*n* = 1), recurrent tumor growth (*n* = 2), and new primary tumor (*n* = 1).

The study was stopped prematurely due to the low inclusion rate and high attrition rate.

No significant differences regarding the change in mouth opening between the two stretching devices were found. Patients who exercised with the TheraBite gained 3.0 mm (IQR − 2.0; 4.0) and those who exercised with the DTS gained 1.5 mm (1.0; 3.0) (*p* = 0.628). Patients who started exercises 36 months or less after tumor treatment gained 2.5 mm (IQR 1.0; 3.0) and those who started more than 36 months after tumor treatment 2.0 mm (IQR − 2.0; 4.0) (*p* = 0.936). One patient recovered from trismus. This patient, who received the TheraBite, had an MMO of 33 mm on T1 and 38 mm on T2 and T3. Additional data can be found in Supplementary Material Tables 1–3.

During the interview, five patients stated that they experienced a gain in mouth opening immediately after exercises, but it declined soon thereafter. Two patients reported a gain in mouth opening during the first weeks (approximately 4 weeks), but no further gain thereafter. Three patients reported no effect even though they complied with the protocol regimen. One patient reported more saliva flow in the front part of the mouth, four patients reported improved suppleness of the mouth (which led to improvements in speaking and eating), one patient reported improved smell and taste, but was in doubt whether it was related to the stretching exercises. Two patients who received the TheraBite reported difficulties with using the device due to insufficient grip as the plates became slippery and due to hampered movement as the rotation angle of the lower plate and the lower jaw were not the same. One patient who received the DTS reported difficulty applying enough force, as the upper denture tilted when force was applied.

## Discussion

No significant effects were found between the stretching devices regarding mouth opening. Other studies reported greater effects after stretching; 5.4 mm (SD 5.7) using the TheraBite [5] or 6.2 mm (SD 3.4) using the DTS [6]. Both studies were retrospective in the design, which might have led to selection bias and an overestimation of the effect. Additionally, both of these studies had a higher frequency of follow-up appointments at shorter intervals, during which patients were informed, guided, and motivated, which might have led to improved compliance and better execution of exercises [5, 6].

Our high attrition rate (56%) and subsequently our small sample size might have been too small to detect the effects of the two stretching devices. High attrition rates are common in similar studies, ranging from 11 to 42% [6–11]. Most common reasons for drop-out are either related to other patient factors, such as mortality [6–9, 12], tumor progression (recurrent tumor or metastasis) [7, 8, 12], or lack need for treatment [7] or are related to stretching problems, such as pain during exercises [8, 10, 11], no perceived improvement [6], and fitting issues of stretching device [7, 10]. In future studies these factors influencing the progression of the stretching protocol need to be taken into account.

## Conclusion

No differences in effects between the TheraBite and DTS were found in our study population. High attrition rates and stretching problems are common when prescribing an intensive stretching protocol on head and neck cancer patients.

### Data

The corresponding author has full control of all primary data. Primary data is available on request.

## Electronic supplementary materials

ESM 1 (DOCX 21 kb)

ESM 2 (DOCX 14 kb)

ESM 3 (DOCX 21 kb)

ESM 4 (DOCX 23 kb)

ESM 5 (DOCX 21 kb)
