# Supplementary material for: The use of stretching devices for treatment of trismus in head and neck cancer patients: a randomized controlled trial
Source: Support Care Cancer. 2019 Nov 7;28(1):9–11. doi: 10.1007/s00520-019-05075-7 (PMC6892373; doi:10.1007/s00520-019-05075-7)
Supplement: Supplementary file 1 — (DOCX 21 kb) [file 520_2019_5075_MOESM1_ESM.docx]

Supplementary Text 1. Further specification of Materials and Methods.

Effects of stretching devices on trismus in head and neck cancer patients: a randomized controlled trial.
Supportive care in cancer.
Sarah J. van der Geer, DMD^1;^ Harry Reintsema, DMD, PhD^1^;Jolanda.I. Kamstra, MD, DMD, PhD^1^;Jan L.N. Roodenburg, DMD, PhD^1^;Pieter U. Dijkstra, PT, PhD^1,2^.
1. Department of Oral and Maxillofacial Surgery, University of Groningen, University Medical Center Groningen, Hanzeplein 1, 9713 GZ, Groningen, the Netherlands
2. Department of Rehabilitation, University of Groningen, University Medical Center Groningen, Hanzeplein 1, 9713 GZ, Groningen, the Netherlands
s.j.van.der.geer@umcg.nl

This trial was registered on 5 December 2015, NTR (Dutch Trial Register) 5589. The study was approved by the Medical Ethical Committee of the University Medical Center Groningen (METC number 2015.468).

Study Population
Patients were recruited from March 2016 till December 2017 at the Department of Oral and Maxillofacial Surgery at the University Medical Center Groningen (UMCG). Patients were checked for eligibility when they visited the Department of Oral and Maxillofacial Surgery during the recruitment period and a restricted mouth opening was observed or reported in the patient file during previous visits. Patients were also assessed for eligibility after responding to a recruitment poster in the waiting area.
Patients were excluded from the study if they were not diagnosed with head and neck cancer, were unable to provide informed consent, were diagnosed with osteoporosis, osteonecrosis or osteoradionecrosis, had severe periodontitis or mobility of teeth, had an oral abscess or other infectious process in the head and neck region, or had a tumor recurrence, metastasis or a new primary tumor in the head and neck region.

Patients who met the inclusion criteria were informed about the study, and their informed consent was obtained prior to enrollment.

Sample size calculation
A maximal mouth opening (MMO) difference of 5 millimeters between the two stretching devices was considered clinically relevant. We assumed a within group standard deviation of 6 mm.[1] With an alpha of 0.05 and a power of 80%, we calculated the sample size using the PS Power and Sample Size Calculations, Version 3.0. In total, 24 subjects per group were needed. To compensate for drop-outs, we aimed to include 30 patients per group.

Randomization
After informed consent was obtained, the researcher (JG) enrolled the patients in the study. JG provided the necessary information for randomization (name, date of birth, interval between last tumor treatment and exercise protocol) to a secretary at the department of Rehabilitation.

The secretary recorded the data, opened an opaque envelope (concealed) containing the assigned stretching device, and recorded the assigned stretching device on a log sheet. The secretary then scheduled appointments for the patients for the assigned intervention.

Blinding
The patient, researcher and therapists (physical therapist or dentist) could not be blinded due to logistic reasons.

The exercise program
The TheraBite exercise program consisted of two options: 20 stretches per session, 6 times a day, 30 seconds per stretch, or 30 stretches per session, 4 times a day, 30 seconds per stretch. The DTS exercise program consisted of 1 stretch per session, 3 times a day, 30 minutes per stretch.
Patients performed the exercises for 3 months. During the first visit (T1), the stretching device and a diary were provided by the therapists. The diary was meant for registration of exercise compliance, pain before, during and after exercises (using the VAS score), intake of pain medication, and pain relief in the form of heat or cold application. The TheraBite exercise program was provided by a physical therapist and the DTS exercise program was provided by a dentist. The patients filled in questionnaires (Mandibular Functional Impairment Questionnaire (MFIQ)[2], European Organization for Research and Treatment of Cancer Quality of Life Questionnaire Cancer module (EORTC QLQ-C30), European Organization for Research and Treatment of Cancer Quality of Life Questionnaire Head & Neck cancer modules (EORTC QLQ-H&N35) [3-5]) and their MMO was measured.

To evaluate stretching exercises and to answer questions, the patients visited the therapists after 3 and 6 weeks. Due to the high burden of the program for patients, the visits after 3 and 6 weeks were replaced by telephone consultations. During the second visit (T2), 12 weeks after T1, the stretching device and diary were handed in. The patients filled in questionnaires (MFIQ, EORTC QLQ-C30, EORTC QLQ-H&N35 and the Dutch version of the Quebec User Evaluation of Satisfaction with Assistive Technology questionnaire 2.0 (D-Quest 2.0) [6] ) and MMO was measured. During the third visit (T3), 26 weeks after T1, the patients filled in questionnaires (MFIQ, EORTC QLQ-C30 and EORTC QLQ-H&N35) and MMO was measured again.
During the visits, JG had an unstructured interview with the patients about their experiences with the stretching devices and exercise protocol.

Data collection
Additional patient data was retrieved from the patient files in the hospital information system: sex, date of birth, tumor localization (maxilla or mandible, tongue, cheek, pharynx, salivary glands, others), cT classification based on the Union for International Cancer Control (UICC) TNM classification 2009 (T1-2, T3-4, no classification available), date of last cancer treatment, surgery (yes, no), neck dissection (yes, no), reconstruction after surgery (skin graft, soft tissue flap, bony tissue flap), radiotherapy (yes, no), total dose of radiotherapy (Gy), and chemotherapy (yes, no).

For the primary outcome, MMO was measured by JG using a sliding caliper. Patient ID, MMO and dental status were recorded on a separate form. Patients were classified as dentate when they had frontal dentition or wore prosthesis. The incisal edges of the upper central incisor 11 and the lower central incisor 41 were used as measurement points. Patients were classified as edentulous if they had no frontal dentition and wore no prosthesis. The top of the alveolar ridge at the former location of the 11 and 41 were used as measurement points. Patients were classified as partially edentulous if they had a frontal dentition or wore prosthesis in one jaw (upper or lower jaw) and had no dentition or wore no prosthesis on the other jaw (upper or lower jaw).

For the secondary outcomes, the questionnaires and the diary were collected.

Statistical analysis
Due to the small sample sizes and skewed data, non- parametric analyses were performed. MMO measurements and the scores of the domains of the questionnaires were described using the median and interquartile range. The differences between the groups (TheraBite and DTS) and the differences in changes over time between the groups regarding MMO, MFIQ and EORTC QLQ-H&N35 question “Opening the mouth”were analyzed using the Mann-Whitney U test. The differences within groups were analyzed using the Friedman test, or Wilcoxon signed-rank test. P-values less than 0.05 were considered statistically significant. Analyses were performed using IBM SPSS Statistics Program version 23.0.

References

1. Dijkstra PU, Sterken MW, Pater R, Spijkervet FK, Roodenburg JL (2007) Exercise therapy for trismus in head and neck cancer. Oral Oncol 43:389-394

2. Stegenga B, de Bont LG, de Leeuw R, Boering G (1993) Assessment of mandibular function impairment associated with temporomandibular joint osteoarthrosis and internal derangement. J Orofac Pain 7:183-195

3. Bjordal K, de Graeff A, Fayers PM, Hammerlid E, van Pottelsberghe C, Curran D, Ahlner-Elmqvist M, Maher EJ, Meyza JW, Bredart A, Soderholm AL, Arraras JJ, Feine JS, Abendstein H, Morton RP, Pignon T, Huguenin P, Bottomly A, Kaasa S (2000) A 12 country field study of the EORTC QLQ-C30 (version 3.0) and the head and neck cancer specific module (EORTC QLQ-H&N35) in head and neck patients. EORTC Quality of Life Group. Eur J Cancer 36:1796-1807

4. Bjordal K, Hammerlid E, Ahlner-Elmqvist M, de Graeff A, Boysen M, Evensen JF, Biorklund A, de Leeuw JR, Fayers PM, Jannert M, Westin T, Kaasa S (1999) Quality of life in head and neck cancer patients: validation of the European Organization for Research and Treatment of Cancer Quality of Life Questionnaire-H&N35. J Clin Oncol 17:1008-1019

5. Singer S, Wollbruck D, Wulke C, Dietz A, Klemm E, Oeken J, Meister EF, Gudziol H, Bindewald J, Schwarz R (2009) Validation of the EORTC QLQ-C30 and EORTC QLQ-H&N35 in patients with laryngeal cancer after surgery. Head Neck 31:64-76

6. Wessels RD, De Witte LP (2003) Reliability and validity of the Dutch version of QUEST 2.0 with users of various types of assistive devices. Disabil Rehabil 25:267-272
