# Supplementary material for: The use of stretching devices for treatment of trismus in head and neck cancer patients: a randomized controlled trial
Source: Support Care Cancer. 2019 Nov 7;28(1):9–11. doi: 10.1007/s00520-019-05075-7 (PMC6892373; doi:10.1007/s00520-019-05075-7)
Supplement: Supplementary file 2 — (DOCX 14 kb) [file 520_2019_5075_MOESM2_ESM.docx]

Supplementary Text 2. Description of occurred adverse events.

Effects of stretching devices on trismus in head and neck cancer patients: a randomized controlled trial.
Supportive care in cancer.
Sarah J. van der Geer, DMD^1;^ Harry Reintsema, DMD, PhD^1^;Jolanda.I. Kamstra, MD, DMD, PhD^1^;Jan L.N. Roodenburg, DMD, PhD^1^;Pieter U. Dijkstra, PT, PhD^1,2^.
1. Department of Oral and Maxillofacial Surgery, University of Groningen, University Medical Center Groningen, Hanzeplein 1, 9713 GZ, Groningen, the Netherlands
2. Department of Rehabilitation, University of Groningen, University Medical Center Groningen, Hanzeplein 1, 9713 GZ, Groningen, the Netherlands
s.j.van.der.geer@umcg.nl

One patient suffered from extreme migraine attacks and vision difficulties during the exercise program. This patient was familiar with a history of migraine attacks and vision difficulties before the exercise program began. In consultation with an ophthalmologist, it was determined that the migraines and vision difficulties were not triggered by the stretching exercises.
Two patients had ulcers related to wearing dental prosthesis. If an ulcer was observed, the prosthesis was adjusted. Three patients had sores in the corner of their mouths; petroleum jelly was advised and was in most cases a sufficient treatment. Two patients reported problems with tenderness of the masticatory muscles, which was related to the exercises. Patients used pain medication, hot or cold application, reduced force of stretching device, or reduced intensity of exercise protocol.
One patient had an uncomplicated fracture of a molar. The sharp edges were removed. Another patient had jaw spasms during and after stretching, due to incorrect use of the stretching device. Together with the physical therapist, the use of the stretching device was modified.
One patient experienced pain while stretching. A depression developed and exercises were stopped.
