# Supplementary material for: The use of stretching devices for treatment of trismus in head and neck cancer patients: a randomized controlled trial
Source: Support Care Cancer. 2019 Nov 7;28(1):9–11. doi: 10.1007/s00520-019-05075-7 (PMC6892373; doi:10.1007/s00520-019-05075-7)
Supplement: Supplementary file 3 — (DOCX 21 kb) [file 520_2019_5075_MOESM3_ESM.docx]

Supplementary Table 1. Patient, tumor and treatment characteristics of patients receiving TheraBite versus DTS and patients who discontinued the study versus those who completed the study.

Effects of stretching devices on trismus in head and neck cancer patients: a randomized controlled trial.
Supportive care in cancer.
Sarah J. van der Geer, DMD^1;^ Harry Reintsema, DMD, PhD^1^;Jolanda.I. Kamstra, MD, DMD, PhD^1^;Jan L.N. Roodenburg, DMD, PhD^1^;Pieter U. Dijkstra, PT, PhD^1,2^.
1. Department of Oral and Maxillofacial Surgery, University of Groningen, University Medical Center Groningen, Hanzeplein 1, 9713 GZ, Groningen, the Netherlands
2. Department of Rehabilitation, University of Groningen, University Medical Center Groningen, Hanzeplein 1, 9713 GZ, Groningen, the Netherlands
s.j.van.der.geer@umcg.nl

|  | **TheraBite**  **(n=14)** | **DTS  (n=13)** | *p* | **Discontinued (n=15)** | **Complete**  **(n=12)** | *p* |
| --- | --- | --- | --- | --- | --- | --- |
| Patient characteristics |  |  |  |  |  |  |
| Male | 7(50) | 8(62) | *0.547* | 6(40) | 9(75) | *0.069^c^* |
| Age at baseline (years) | 67.8(63.6;73.9) | 64.7(56.3;69.1) | *0.140^a^* | 68.0(62.4;75.3) | 64.0(57.9;68.5) | *0.166^a^* |
| Maximal mouth opening at baseline (millimeters) | 27.0(18.0;29.0) | 22.0(19.0;25.0) | *0.663^a^* | (21.5(14.5;28.0) | (24.0(20.5;28.5) | *0.235^a^* |
| Dental status^d^ |  |  | *0.825* |  |  | *0.385* |
| Dentulous | 10(77) | 8(73) |  | 8(67) | 10(83) |  |
| Partially edentulous | 2(15) | 1(9) |  | 1(8) | 2(17) |  |
| Edentulous | 1(8) | 2(18) |  | 3(25) | 0(0) |  |
| Interval between last tumor treatment and start exercise protocol (months) |  |  |  |  |  |  |
| Group ≤36 months | 8 (57) | 8 (62) |  | 8(57) | 7(54) |  |
| Group ≤36 months | 16.0(8.1;16.7) | 9.7(7.2;12.6) | *0.418^a^* | 9.3(8.3;11.2) | 15.0(8.1;16.1) | *0.556^a^* |
| Group >36 months | 6 (43) | 5 (39) |  | 6(43) | 6(46) |  |
| Group >36 months | 98.4(54.2;132.7) | 53.2(41.0;157.0) | *0.439^a^* | 119.3(54.2;132.7) | 65.3(42.5;157.0) | *0.606^a^* |
| Tumor characteristic |  |  |  |  |  |  |
| Multiple primary tumors | 2(14) | 4(31) | *0.385* | 4(27) | 2(17) | *0.662* |
| Tumor localization |  |  | *0.840* |  |  | *0.558* |
| Maxilla or Mandible | 4(29) | 2(15) |  | 2(13) | 4(33) |  |
| Tongue | 1(7) | 3(23) |  | 3(20) | 1(8) |  |
| Cheek | 1(7) | 2(15) |  | 1(7) | 2(17) |  |
| Pharynx | 5(36) | 3(23) |  | 4(27) | 4(33) |  |
| Salivary glands | 1(7) | 1(8) |  | 2(13) | 0(0) |  |
| Others | 2(14) | 2(15) |  | 3(20) | 1(8) |  |
| cT-stage^d^ |  |  | *0.188* |  |  | *1.000* |
| cT1-2 | 5(36) | 3(23) |  | 4(27) | 4(33) |  |
| cT3-4 | 9(64) | 7(54) |  | 9(60) | 7(58) |  |
| Treatment characteristics |  |  |  |  |  |  |
| Surgery | 10(71) | 10(77) | *1.000* | 11(73) | 9(75) | *1.000* |
| Neck dissection | 8(57) | 7(54) | *1.000* | 7(50) | 8(67) | *0.391* |
| Reconstruction |  |  | *0.571* |  |  | *0.267* |
| Skin graft | 1(20) | 3(60) |  | 2(100) | 2(25) |  |
| Soft tissue flap | 2(40) | 1(20) |  | 0(0) | 3(38) |  |
| Bony tissue flap | 2(40) | 1(20) |  | 0(0) | 3(38) |  |
| Radiotherapy | 6(43) | 5(39) | *1.000* | 9(60) | 2(17) | ***0.047^b^*** |
| Total dose (Gy) | 70.0(60.0;70.0) | 68.0(66.0;70.0) | *0.739^a^* | 70.0(59.0;70.0) | 68.0(66.0;70.0) | *0.970^a^* |
| Chemotherapy | 4(29) | 5(39) | *0.695* | 3(20) | 6(50) | *0.217* |
| TheraBite |  |  |  | 8(53) | 6(50) | *0.863* |
| DTS |  |  |  | 7(47) | 6(50) |  |
| Heat or cold application | 3(21) | 2(15) | *1.000* | 3(20) | 2(17) | *1.000* |
| Intake pain medication | 3(21) | 1(8) | *0.596* | 2(13) | 2(17) | *1.000* |
| Continue exercising after 3 months | 2(33) | 3(38) | *1.000* | 1(50) | 4(33) | *1.000* |

Data is presented in numbers (%) or median (25th;75th percentile)
P-values are the result of the chi square test.
a: p-values are the result of the Mann Whitney-U test.
b: p-values <0.05
c: p-values near significance level (<0.05)
d: Dental status: data of three patients are missing, these patients dropped out of the study before measurement could take place. cT-classification: data of three patients are missing, as information could not be retrieved from the patient files in the hospital information system.
