# Supplementary material for: The use of stretching devices for treatment of trismus in head and neck cancer patients: a randomized controlled trial
Source: Support Care Cancer. 2019 Nov 7;28(1):9–11. doi: 10.1007/s00520-019-05075-7 (PMC6892373; doi:10.1007/s00520-019-05075-7)
Supplement: Supplementary file 4 — (DOCX 23 kb) [file 520_2019_5075_MOESM4_ESM.docx]

Supplementary Table 2. Differences between groups (TheraBite versus DTS) at T1,T2, and T3.

Effects of stretching devices on trismus in head and neck cancer patients: a randomized controlled trial.
Supportive care in cancer.
Sarah J. van der Geer, DMD^1;^ Harry Reintsema, DMD, PhD^1^;Jolanda.I. Kamstra, MD, DMD, PhD^1^;Jan L.N. Roodenburg, DMD, PhD^1^;Pieter U. Dijkstra, PT, PhD^1,2^.
1. Department of Oral and Maxillofacial Surgery, University of Groningen, University Medical Center Groningen, Hanzeplein 1, 9713 GZ, Groningen, the Netherlands
2. Department of Rehabilitation, University of Groningen, University Medical Center Groningen, Hanzeplein 1, 9713 GZ, Groningen, the Netherlands
s.j.van.der.geer@umcg.nl

|  | **TheraBite** | **DTS** | **T1** | **TheraBite** | **DTS** | **T2** | **TheraBite** | **DTS** | **T3** |
| --- | --- | --- | --- | --- | --- | --- | --- | --- | --- |
|  | Med (IQR) | Med (IQR) | *p* | Med (IQR) | Med (IQR) | *p* | Med (IQR) | Med (IQR) | *p* |
|  |  |  |  |  |  |  |  |  |  |
| **Maximal mouth opening (millimeters)** | 27.0 (18.0;29.0) | 22.0 (19.0;25.0) | *0.663* | 25.0  (16.0;32.0) | 26.0 (22.0;28.0) | *0.849* | 29.0  (25.0;32.0) | 24.5 (17.0;30.0) | *0.261* |
| **MFIQ** | 34.0 (26.3;38.6) | 28.0 (13.0;37.0) | *0.185* | 31.0  (27.0;42.0) | 15.5 (9.0;36.0) | *0.200* | 31.5 (27.5;37.0) | 8.3 (5.0;17.0) | *0.065^a^* |
| **EORTC QLQ c30** |  |  |  |  |  |  |  |  |  |
| Global quality of life | 83.3 (75.0;83.3) | 83.3 (75.0;100.0) | *0.276* | 83.3 (66.7;91.7) | 91.7 (75.0;100.0) | *0.456* | 66.7 (58.3;83.3) | 87.5 (75.0;100.0) | *0.197* |
| Physical Functioning | 90.0 (73.3;100.0) | 86.7 (80.0;100.0) | *0.750* | 80.0  (66.7;93.3) | 86.7 (60.0;100.0) | *0.560* | 90.0 (66.7;100.0) | 96.7 (86.7;100.0) | *0.616* |
| Role Functioning | 83.3 (58.3;100.0) | 100.0 (83.3;100.0) | *0.101* | 83.3 (50.0;100.0) | 100.0 (100.0;100.0) | *0.315* | 83.3 (66.7;100.0) | 100.0 (100.0;100.0) | *0.209* |
| Emotional Functioning | 100.0 (83.3;100.0) | 91.7 (58.3;100.0) | *0.153* | 95.8  (83.3;100.0) | 100.0 (75.0;100.0) | *0.820* | 83.3 (75.0;100.0) | 100.0 (91.7;100.0) | *0.231* |
| Cognitive Functioning | 91.7 (66.7;100.0) | 100.0 (83.3;100.0) | *0.713* | 100.0 (66.7;100.0) | 100.0 (83.3;100.0) | *0.777* | 91.7 (83.3;100.0) | 100.0 (100.00;100.00) | *0.293* |
| Social Functioning | 100.0 (91.7;100.0) | 100.0 (66.7;100.0) | *0.495* | 100.0  (66.7;100.0) | 100.0 (100.0;100.0) | *0.916* | 75.0 (66.7;83.3) | 100.0 (100.0;100.0) | *0.072* |
| Fatigue | 11.1 (0.0;38.9) | 16.7  (0.0;55.6) | *0.801* | 22.2 (0.0;33.3) | 11.1 (0.0;11.1) | *0.443* | 33.3 (22.2;44.4) | 5.6 (0.0;11.1) | *0.140* |
| Nausea and vomiting | 0.0 (0.0;0.0) | 0.0 (0.0;0.0) | *0.563* | 0.0  (0.0;0.0) | 0.0 (0.0;0.0) | *0.909* | 0.0 (0.0;33.3) | 0.0 (0.0;16.7) | *0.924* |
| Pain | 0.0 (0.0;16.7) | 0.0 (0.0;33.3) | *0.744* | 8.3  (0.0;33.3) | 0.0 (0.0;50.0) | *0.785* | 16.7  (0.0;33.3) | 0.0 (0.0;16.7) | *0.388* |
| Dyspnea | 0.0 (0.0;33.3) | 0.0 (0.0;0.0) | *0.183* | 0.0  (0.0;33.3) | 0.0 (0.0;0.0) | *0.426* | 16.7 (0.0;33.3) | 0.0 (0.0;0.0) | *0.211* |
| Insomnia | 0.0 (0.0;16.7) | 0.0 (0.0;33.3) | *0.904* | 0.0 (0.0;33.3) | 0.0 (0.0;33.3) | *0.849* | 33.3  (0.0;33.3) | 0.0 (0.0;0.0) | *0.083* |
| Appetite loss | 0.0 (0.0;0.0) | 0.0 (0.0;0.0) | *0.922* | 0.0  (0.0;0.0) | 0.0 (0.0;33.3) | *0.398* | 16.7 (0.0;33.3) | 0.0 (0.0;0.0) | *0.386* |
| Constipation | 0.0 (0.0;16.7) | 0.0 (0.0;66.7) | *0.245* | 0.0 (0.0;33.3) | 0.0 (0.0;0.0) | ***0.039**** | 0.0  (0.0;33.3) | 0.0 (0.0;0.0) | *0.673* |
| Diarrhea | 0.0 (0.0;16.7) | 0.0 (0.0;0.0) | *0.325* | 0.0  (0.0;0.0) | 0.0 (0.0;0.0) | *0.699* | 0.0 (0.0;0.0) | 0.0 (0.0;0.0) | *1.0000* |
| Financial difficulties | 0.0 (0.0;0.0) | 0.0 (0.0;33.3) | *0.225* | 0.0 (0.0;0.0) | 0.0 (0.0;0.0) | *0.818* | 0.0  (0.0;0.0) | 0.0 (0.0;33.3) | *0.673* |
| **EORTC QLQ H&N35** |  |  |  |  |  |  |  |  |  |
| Pain | 20.8 (16.7;33.3) | 25.0 (8.3;41.7) | *0.803* | 29.2 (22.5;33.3) | 25.0 (0.0;33.3) | *0.708* | 29.2 (16.7;41.7) | 16.7 (0.0;25.0) | *0.294* |
| Swallowing | 16.7 (8.3;25.0) | 8.3 (0.0;16.7) | *0.056^a^* | 16.7 (0.0;25.0) | 8.3 (0.0;8.33) | *0.446* | 20.8 (8.3;41.7) | 0.0 (0.0;8.3) | *0.081* |
| Problems with senses | 25.0 (0.0;33.3) | 0.0 (0.0;0.0) | ***0.009**** | 16.7  (0.0;50.0) | 0.0 (0.0;0.0) | *0.055^a^* | 33.3 (33.3;33.3) | 0.0 (0.0;0.0) | ***0.034**** |
| Problems with speech | 11.1 (0.0;38.9) | 0.0 (0.0;11.1) | *0.341* | 0.0 (0.0;55.6) | 0.0 (0.0;11.1) | *0.706* | 16.7  (11.1;33.3) | 0.0 (0.0;11.1) | *0.132* |
| Trouble with social eating | 33.3 (12.5;37.5) | 8.3 (0.0;50.0) | *0.289* | 33.3 (16.7;50.0) | 8.3 (0.0;58.3) | *0.284* | 20.8 (16.7;41.7) | 4.2 (0.0;8.3) | *0.051^a^* |
| Trouble with social contact | 6.7  (0.0;18.3) | 0.0  (0.0;6.7) | *0.405* | 0.0  (0.0;13.33) | 0.0 (0.0;13.3) | *0.925* | 6.7  (6.7;13.3) | 0.0 (0.0;0.0) | *0.101* |
| Less sexuality | 25.0  (0.0;33.3) | 0.0 (0.0;66.7) | *0.647* | 25.0 (0.0;58.3) | 0.0 (0.0;100.0) | *0.821* | 41.7 (33.3;66.7) | 0.0 (0.0;0.0) | *0.067^a^* |
| Teeth | 33.3  (16.7;50.00) | 33.3 (0.0;33.3) | *0.204* | 16.7  (0.0;100.0) | 33.3 (0.0;33.0) | *0.693* | 16.7  (0.0;66.7) | 0.0(0.0;33.3) | *0.367* |
| Opening mouth | 83.3 (50.0;100.0) | 33.3 (33.3;100.0) | *0.327* | 66.7 (33.3;100.0) | 33.3  (0.0;66.7) | *0.083* | 66.7  (33.3;66.7) | 33.3 (33.3;66.7) | *0.309* |
| Dry mouth | 75.0 (50.0;100.0) | 33.3 (0.0;33.3) | ***0.011**** | 83.3 (33.3;100.0) | 0.0 (0.0;33.3) | ***0.009**** | 50.0 (33.3;100.0) | 16.7 (0.0;33.3) | *0.053^a^* |
| Sticky saliva | 66.7 (33.3;83.3) | 0.0 (0.0;33.3) | ***0.006**** | 66.7 (33.3;100.0) | 0.0 (0.0;33.3) | ***0.047**** | 33.3 (33.3;33.3) | 0.0 (0.0;33.3) | *0.227* |
| Coughing | 0.0 (0.0;33.3) | 0.0 (0.0;33.3) | *0.944* | 16.7 (0.0;33.3) | 0.0 (0.0;66.7) | *0.788* | 16.7  (0.0;33.3) | 0.0 (0.0;33.3) | *0.465* |
| Felt ill | 0.0 (0.0;0.0) | 0.0 (0.0;0.0) | *0.950* | 0.0 (0.0;0.0) | 0.0 (0.0;33.3) | *0.673* | 50.0 (0.0;66.7) | 0.0 (0.0;0.0) | *0.105* |
| Pain killers | 0.0 (0.0;100.0) | 0.0 (0.0;100.0) | *0.561* | 100.0 (0.0;100.0) | 0.0 (0.0;100.0) | *0.509* | 50.0 (0.0;100.0) | 50.0 (0.0;100.0) | *1.000* |
| Nutritional supplements | 0.0 (0.0;100.0) | 0.0 (0.0;100.0) | *0.672* | 0.0 (0.0;100.0) | 0.0 (0.0;100.0) | *1.000* | 0.0 (0.0;100.0) | 0.0 (0.0;0.0) | *0.523* |
| Feeding tube | 0.0 (0.0;0.0) | 0.0 (0.0;0.0) | *0.925* | 0.0 (0.0;100.0) | 0.0 (0.0;0.0) | *0.081* | 0.0 (0.0;100.0) | 0.0 (0.0;0.0) | *0.138* |
| Weight loss | 0.0 (0.0;0.0) | 0.0 (0.0;100.0) | *0.547* | 0.0 (0.0;100.0) | 0.0 (0.0;0.0) | *0.331* | 0.0 (0.0;100.0) | 0.0 (0.0;0.0) | *0.523* |
| Weight gain | 0.0 (0.0;0.0) | 0.0 (0.0;0.0) | *0.166* | 0.0 (0.0;0.0) | 0.0 (0.0;0.0) | *0.292* | 0.0 (0.0;0.0) | 0.0 (0.0;0.0) | *0.317* |

P-values are the result of the Mann Whitney-U test.
*: P-values <0.05
a: P-values near significance level (<0.05)
